# Assessment of nurses interventions in the Management of Clinical Alarms in the critical care unit, Kenyatta National Hospital, a cross sectional study

**DOI:** 10.1186/s12912-017-0235-1

**Published:** 2017-07-25

**Authors:** Lucy W. Meng’anyi, Lilian A. Omondi, Margaret N. Muiva

**Affiliations:** 1 Kenyatta National Hospital, P.O. Box 20723, Nairobi, 00202 Kenya; 2 School of Nursing Sciences, University of Nairobi, P.O. Box 19676, Nairobi, 00200 Kenya; 3 Mount Kenya University, Thika, Kenya

**Keywords:** Nursing, Clinical alarms, Intensive care unit, Critical care unit, Management

## Abstract

**Background:**

Alarms in the critical areas are an important component of most of the machines as they alert nurses on the change in the patients’ condition. Most patients in the critical care units cannot speak for themselves hence cannot pinpoint when their condition changes. It is therefore important to assess the nurses’ interventions when managing clinical alarms. The purpose of this study was to assess interventions employed by nurses in the management of clinical alarms in the care of patients in the Critical Care Unit (CCU), Kenyatta National Hospital (KNH).

**Methods:**

A descriptive cross sectional study was carried out in the month of June 2014 where 87 nurses were recruited as study respondents. KNH/ University of Nairobi (UoN) Ethics and Research committee approved the research. A structured self administered questionnaire was used to collect data. The questionnaire contained some questions in a Likert scale in relation to the actions the nurses would take in the management of clinical alarms and some on whether policies on alarm management existed in the hospital, if they filled alarm checklists and how often and the types of alarms they would respond to first.

**Results:**

The respondents’ responses were scored and from the results it was clear that there were some gaps in the management of clinical alarms. Majority of the nurses reported that they respond to alarms of all durations and do not fill alarm checklists as neither alarm checklists nor protocols are provided. From the findings there was a statistically significant association (*p* = 0.06) between age and whether the respondents assessed the cause of the alarm beep.

**Discussion:**

Respondents in this study respond to alarms of all durations in contrast to other studies where the findings indicate that nurses respond to alarms for different reasons, not just that the alarm sounds. Majority of the respondents scored averagely on the questions on whether they carry out most of the interventions or actions. This is inline with previous studies which have shown that healthcare personnel respond to alarms depending on the patient’s physiological status.

**Conclusions:**

Nurses in the unit carry out the standard nursing interventions on clinical alarms and, respond to alarms of all durations and do not fill alarm checklists. Alarm protocols should therefore be developed in the hospital, the nurses should be trained on management of clinical alarms and more nurses employed.

**Electronic supplementary material:**

The online version of this article (doi:10.1186/s12912-017-0235-1) contains supplementary material, which is available to authorized users.

## Background

Perceived alarm urgency contributes to the nurse’s alarm response but nurses use additional strategies to determine response including the criticality of the patient, signal duration, rarity of alarming device and workload. A caregiver shall respond to an alarm if he or she perceives it to be true. “If an alarm system is perceived to be 90% reliable, the response rate shall be about 90%, if the alarm system is perceived to be 10% reliable, the response rate shall be about 10%. Nurses respond to alarms for different reasons, not just the fact that the alarm sounds [1].”

Various devices in the CCU have alarms whose various goals are: to detect life threatening situations, detect imminent danger, diagnose (diagnostic alarms, indicate a pathophysiological condition e.g. shock), detection of life threatening device malfunction e.g. disconnection from the patient, occlusion of the connection to the patient, disconnection from power, gas etc. and detection of imminent device malfunctions [2].

Nurses adjust the order of their activities by evaluating alarm urgency in relation to the patients’ condition and have a greater tendency to react to alarms of longer duration and considered rare,. As workload complexity increases, alarm response and task performance deteriorates. Thus signal duration is an important influence to the nurses’ response but workload, patient condition and task complexity may lead to other reaction strategies. Adjusting alarms to patient’s actual needs ensures that alarms are valid and provides an early warning to potential critical situations [1].

Nurses are at risk of becoming desensitized to the alarms that are meant to protect patients when the frequency of alarms is high. Nurses in the CCUs in the Northeastern academic medical center in the USA stated that the primary problem with alarms is that they are continuously alarming and that the largest contributor to the number of false alarms in the CCUs is the pulse oximetry alarm [3].

According to the American Association of Critical-Care Nurses (AACN, 2012) practice alerts, alarm fatigue develops when a person is exposed to an excessive number of alarms of which most could be false alarms. This may result in sensory overload, which may cause the person to become desensitized to the alarms [4]. Patient deaths have been attributed to alarm fatigue [4]. The AACN therefore has suggested several strategies to improve patient safety in the event of reducing the number of false alarms [5].

The strategies recommended by AACN are: proper skin preparation for Electrocardiogram (ECG) electrodes by; washing the isolated electrode area with soap and water to decrease skin impedance and signal noise thereby enhancing conductivity, wiping the electrode with a rough washcloth or gauze and or using sandpaper on the electrode to roughen a small area of the skin to prevent spurious signals which are recorded when there is poor electrode contact and it also helps to remove part of the stratum corneum to allow the electrical signals to travel. Other strategies are; daily change of electrodes, customization of delay and threshold settings on oxygen saturation via pulse oximetry machines, use of disposable pulse oximetry sensors and replacing the sensors when they no longer adhere properly to the patient’s skin. Alcohol should not be used for skin preparation as it can dry out the skin. Excessive hair at the electrode site should also be clipped [5].

The AACN also recommends that institutions should provide initial and ongoing education about devices with alarms. The education should be on monitoring systems and alarms as well as operational effectiveness to new nurses and all other health care staff on a periodic basis [5].

Evidence suggests that daily changing of electrodes decreases the number of false alarms. In a quality improvement study in the United States of America (USA), the average percentage of alarms per bed decreased by 46% by changing ECG electrodes daily [5].

Changing alarm default settings and customizing alarms according to patient need, including parameters and levels, have decreased the number of false alarms in some institutions. A 43% reduction in critical care alarms was observed in a CCU setting in USA, when default alarm parameters were changed and registered nurses were educated about the change. Similarly in a medical-surgical unit with telemetry monitoring, changing the high heart rate alarm from 120 beats per minute (bpm) to 130 bpm resulted in a 50% decrease in the number of alarms [5].

The aim of this research was to determine the various interventions employed by nurses in the management of clinical alarms in the care of critically ill patients at the CCU, KNH (KNH records). The data bases for literature review were obtained from: Hinari, Pubmed, Google scholar.

## Methods

This was a descriptive cross sectional quantitative study conducted in the month of June 2014 whereby 87 nurses in the CCU, KNH were recruited as study respondents. All the respondents consented to participate in the study. Approval to conduct the study was sought from KNH/UoN Ethics Committee and funds provided by KNH. A self administered questionnaire was used to collect data on: socio demographic factors, general questions about alarms and the various interventions employed by nurses in the management of clinical alarms in the care of critically ill patients. The questionnaire was pretested at the KNH Acute Room, Accident and Emergency Unit on nurses. This was an ideal unit to pretest the tool as the alarm limit settings in the clinical device systems in the acute room are the same as the settings in the CCU. The nurses in the acute room take care of critically ill patients just like the CCU as they are mostly on transit into the CCU or theatre. The researcher was therefore able to review and amend questions that were not clearly understood by respondents hence ensuring validity and reliability of the instrument.

The sample size of 87 respondents was determined using the Cochran’s formula [6] and since the target population was less than 10,000, the Fisher et al. formula [7] was used to give a further sample size estimate.

Convenience sampling method was used to recruit study respondents because of the different work shifts. The inclusion criteria was: all qualified nurses who had worked in the CCU for 6 months and more and permanently deployed to the unit, all qualified nurses who were in the unit at the time of the study but not permanently deployed to the unit and consented to participate in the study. The exclusion criteria was: all staff in the CCU who were not nurses- the doctors, biomedical staff and the support staff, student nurses who were on clinical rotation in the unit, nurses who declined to participate in the study and nurses who were absent at the time of data collection.

Independent variables were: age, gender and professional qualifications of the respondents, and experience of the respondents which was measured by the number of years the respondents had worked in the unit. Dependent variables were: nurses’ responses or the actions the nurses took when the clinical alarms set off.

SPSS version 20 [8] was used to analyze data. Chi square (X^2^) and Cramer’s V (*P* = ≤0.05) were used to establish the relationship between the independent variables and the nurses’ responses to alarms.

## Results

### Alarms that the nurses are more likely to respond to

**Fig. 1:**
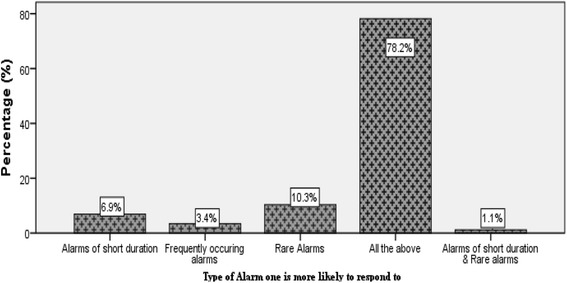
Alarms that Nurses are more likely to respond to

As presented in Fig. 1, majority of the nurses 68 (78.2%) reported that they were likely to respond to alarms of all durations. A few 9 (10.3%) were more likely to respond to rare alarms, 6 (6.9%) alarms of short duration, 3 (3.4%) frequently occurring alarms and1 (1.1%) alarms of short duration and rare alarms.

### Number of respondents that fill alarm checklists and the frequency of filling alarm checklists

Most 71 (81.6%) of the respondents did not fill alarm checklists while only 16 (18.4%) reported filling alarm checklists (Table 1).

**Table 1:** Number that fill alarm checklists and how often

Variable	Frequency (n)	Percentage (%)
How many fill in alarm checklists		
Yes	16	18.4
No	71	81.6
If they fill in alarm checklists, how often		
Daily	6	6.9
At the start of the shift	6	6.9
Occasionally	4	4.6
During admission	-	-

### Reasons given by the respondents as to why they do not fill alarm checklists

A total of 55 (100%) nurses responded to this question and of this 5.5% reported that no alarm checklists have been provided in the unit and there are no protocols stating that they have to fill alarm checklists and 94.5% gave other reasons as to why they do not fill alarm checklists as shown in Table 2.

**Table 2:** Reasons as to why the respondents do not fill alarm checklists

Reason	Frequency (n)	Percentage (%)
1. No alarm checklists have been provided in the unit and there are no protocols stating that they have to fill alarm checklists Reasons: • The issue had not been highlighted in the unit • The checklists are out of stock • Monitors record the events of the alarms	3	5.5
2. Other reasons as to why they do not fill alarm checklists • They do not have the nursing materials or paperwork • The amount of work in CCU is too much • The alarm trends are indicated in the monitor if need be for retrieving • Has not been considered or thought of in CCU • It is not the norm or practice • They occasionally fill when there is change in patients’ condition • Heavy workload and staff shortage • No provision in the nursing charts • Not provided but I take necessary action • Nurses have not been taught the importance of maintaining alarm checklists • Shortage of monitors or mechanical ventilators and • There is no facilitation for alarm checklist filling.	52	94.5

### Nurses interventions/actions in the management of clinical alarms

Of the respondents, 37 (43%) responded that they: “Always” ensure proper skin preparation of patients before placing electrodes, 67 (77%) “Always” assess the cause of the alarm beep when it alarms, 78 (89.7%) “Never” ignore alarms every time they beep, 61 (70.1%) “Always” check and assess the patient’s condition every time the alarm beeps and 62 (71.3%) “Always” reset the alarm settings of the machine each time they admit a patient (Table 3).

**Table 3:** Frequency and percentages of nurses’ responses to alarms

	Nurse’s response to alarm				
	Variable	Never n (%)	Sometimes n (%)	Often n (%)	Always n (%)
1.	I ensure proper skin preparation of patients before placing electrodes	2 (0.6)	27 (16)	21 (18)	37 (43)
2.	I change the patients’ electrodes daily	5 (2.9)	32 (18.4)	27 (23.3)	23 (26.4)
3.	I assess the cause of the alarm beep when it alarms.	- (0)	6 (3.4)	14 (12.1)	67 (77)
4.	I disable the alarms every time they beep	42 (55.2)	27 (23.3)	11 (6.3)	7 (2)
5.	I pause the alarms every time they beep	21 (24.1)	45 (38.8)	17 (9.8)	4 (1.2)
6.	I reset the alarm limits every time alarms beep	20 (23)	48 (41.4)	10 (5.7)	9 (2.6)
7.	I ignore alarms every time they beep	78 (89.7)	9 (7.8)	-	-
8.	I check and assess the patient’s condition every time the alarm beeps	-	8 (6.9)	18 (10.3)	61 (70.1)
9.	I reset alarm settings of the machines each time I admit a new patient	1 (0.3)	11 (6.3)	13 (11.2)	62 (71.3)

For questions 1,2,3,8 and 9 the grading of the responses is as follows: Never – 1, Sometimes- 2, Often – 3 and Always- 4

For questions 4, 5, 6 and 7, Never – 4, Sometimes- 3, Often – 2 and Always- 1

### Testing of socio demographic characteristics and the action of the nurses: “I ensure proper skin preparation of patients before placing electrodes”

The respondents scored as follows: age group 36–44 years scored 80.7%, Females scored 78.6%, BScN’s scored 82.1%, those nurses who had worked for more than 2 years scored 100%, those nurses who had worked in the CCU for more than 2 years 82.7%, nurses who were CCN trained 80% and those trained in alarm management 84.4%.

### Testing of socio demographic characteristics and the nurses’ action: “I change the patient’s electrodes daily”

The scores were as follows: Age group 45–55 years scored 73.4%, Females 73.4%, MScN’s and KRN’s 100%, those who had worked as nurses for more than 2 years scored 75%, those who had worked in the CCU for more than 10 years 77.9%, those who were CCN trained scored 72.5% and those who had been trained in alarm management scored 81.3%. Generally the respondents did not score very well in this question.

### Testing of socio demographic characteristics and the nurses’ responses to the action: “I disable the alarms every time they beep”

The respondents scored as follows: the age group 25–35 years scored the highest with 81.5%, MScN and KRN scored 100%, those that had worked as nurses for more than 10 years scored 79.8%, those who had worked in CCU for more than 10 years scored 85.6%, those that were CCN trained 80.5%, and those that had undergone alarm management 82.5%.

### Testing of socio demographic characteristics and the nurses’ responses to the action: “I pause every time they beep

The highest scores by the respondents were as follows: age group 25–35 scored 78.7%, males scored 77%, KRNs scored 100%, MScN 75%,, those who had worked as nurses for more than 2 years scored 75%, those who had worked in the CCU department for less than 2 years scored 75%, those that were CCN trained scored 74.4% and those that had been trained in alarm management scored 81.3%.

### Testing of socio demographic characteristics and the nurses’ responses to the action: “I reset the alarm limits every time alarms beep

The age group between 45 and 55 years scored the highest at 75%, males scored 80% and the females 69.4%, the nurses with MScN scored the highest at 75%, those who had worked in CCU for less than 2 years 83.3%, those who had not been trained in critical care nursing 72.5% and those who had been trained in alarm management 75%.

### Testing of socio demographic characteristics and the nurses’ responses to the action: “I assess the cause of the alarm beep when it alarms

The respondents scored as follows in terms of the highest scores: age group 36–44 years 96.6%, females scored 93.2%, KRN 100%, those that had worked as nurses more than 2 years scored 100%, those that had worked in CCU for more than 2 years scored 92.3%, those that were CCN trained scored 92.2% and those that were alarm management trained scored 100%. There was also a statistically significant relationship between the nurses’ age and their response to the action of assessing the cause of the alarm beep (*p* = 0.006) (Tables 4 and 5).

**Table 4:** Association of socio demographic characteristics and the action: “I assess the cause of the alarm beep when it alarms”

Socio demographic variables	Never n (%)	Sometimes n (%)	Often n (%)	Always n (%)	Total score n (%)
Age in years					
25–35		2 (4)	7 (21)	18 (72)	89.8
36–44		0	6 (18)	38 (152)	96.6
45–55		4 (8)	1 (3)	11 (44)	85.9
Gender					
Male		1 (2)	7 (21)	17 (68)	91
Female		5 (10)	7 (21)	50 (200)	93.2
Professional qualification					
KRCHN		6 (18)	11 (33)	61 (244)	94.6
BScN		0	2 (6)	5 (20)	92.9
MScN		0	1 (3)	0	75
KRN		0	0	1 (4)	100
Years worked as a Nurse					
Below 2 years		1 (2)	0	2 (8)	83.3
Above2 years		0	0	2 (8)	100
Above 5 years		0	5 (15)	14 (56)	93.4
Above 10 years		5 (10)	9 (27)	49 (196)	3.7
Years worked in CCU					
Below 2 years		2 (4)	2 (6)	11 (44)	90
Above2 years		1 (2)	2 (6)	10 (40)	92.3
Above 5 years		1 (2)	5 (15)	27 (108)	84.4
Above 10 years		2 (4)	5 (15)	19 (76)	91.3
CCU Trained					
Yes		5 (10)	14 (42)	58 (232)	92.2
No		1 (2)	0	9 (27)	72.5
Alarm management trained					
Yes		0	0	16 (64)	100
No		6 (12)	14 (42)	50 (200)	90.7

**Table 5:** Association of socio demographic characteristics and the action: I assess the cause of the alarm beep when it alarms

Socio demographic variables	Never n (%)	Sometimes n (%)	Often n (%)	Always n (%)	Chi	df	*P* Value	Cramer’s V
Age in years					14.415	4	0.006	0.006
25–35		2 (7.4)	7 (25.9)	18 (66.7)				
36–44		0 (0.0)	6 (13.6)	38 (86.4)				
45–55		4 (25)	1 (6.2)	11 (66.8)				
Gender					3.888	2	0.143	0.143
Male		1 (4)	7 (28)	17 (68)				
Female		5 (8.1)	7 (11.3)	50 (47.7)				
Sampling Technique					6.979	6	0.323	0.323
KRCHN		6 (7.7)	11 (14.1)	61 (78.2)				
BScN		0 (0.0)	2 (28.6)	5 (71.4)				
MScN		0 (0.0)	1 (100)	0 (0.0)				
KRN		0 (0.0)	0 (0.0)	1 (100)				
Years worked as a Nurse					6.965	6	0.324	0.324
Below 2 years		1 (33.3)	0 (0.0)	2 (66.7)				
Above2 years		0 (0.0)	0 (0.0)	2 (100)				
Above 5 years		0 (0.0)	5 (26.3)	14 (73.7)				
Above 10 years		5 (7.9)	9 (14.3)	49 (77.8)				
Years worked in CCU					2.082	6	0.912	0.912
Below 2 years		2 (13.3)	2 (13.3)	11 (73.3)				
Above2 years		1 (7.7)	2 (15.4)	10 (76.9)				
Above 5 years		1 (3)	5 (15.2)	27 (25.4)				
Above 10 years		2 (7.7)	5 (19.2)	19 (73.1)				
CCU Trained					2.223	2	0.329	0.329
Yes		5 (6.5)	14 (18.2)	58 (75.3)				
No		1 (10)	0 (0.0)	9 (90)				
Alarm management trained					5.957	2	0.051	0.051
Yes		0 (0.0)	0 (0.0)	16 (100)				
No		6 (8.6)	14 (20)	50 (71.4)				

### Testing of socio demographic characteristics and the nurses’ responses to the action: “I ignore alarms every time they beep

The respondents responded as follows in terms of the highest scores:

Age group 25–35 years scored 98.2%, there was no much difference between the male and female who scored 96% and 96.8% respectively. MScN and KRN’s scored 100%, the respondents who had worked as nurses for less than 2 years and more than 2 years scored 100%, those who had worked in the CCU for less than two years scored 100%. The respondents who were CCN trained scored 97.5% and the ones who had not been trained in alarm management scored 97.8% (Table 6).

**Table 6:** Association of socio demographic characteristics and the nurses’ action: I ignore alarms every time they beep

Socio demographic variables	Nevern (%)	Sometimes n (%)	Often n (%)	Always n (%)	Total score (%)
Age in years					
25–35	96 (24)	6 (2)		4 (1)	98.2
36–44	160 (40)	12 (4)		0	97.7
45–55	52 (13)	9 (3)		0	95.3
Gender					
Male	84 (21)	12 (4)		0	96
Female	224 (56)	15 (5)		1 (1)	96.8
Professional qualification					
KRCHN	276 (69)	24 (8)		1 (1)	96.5
BScN	24 (6)	3 (1)		0	96.4
MScN	4 (1)			0	100
KRN	4 (1)	0		0	100
Years worked as a Nurse					
Below 2 years	12 (3)	0		0	100
Above2 years	8 (2)	0		0	100
Above 5 years	52 (13)	18 (6)		0	92.1
Above 10 years	236 (59)	9 (3)		1 (1)	97.6
Years worked in CCU					
Below 2 years	60 (15)	0		0	100
Above2 years	36 (9)	12 (4)		0	92.3
Above 5 years	116 (29)	12 (4)		0	97
Above 10 years	96 (24)	3 (1)		1 (1)	96.2
CCN Trained					
Yes	272 (68)	24 (8)		1 (1)	96.4
No	36 (9)	3 (1)		0	97.5
Alarm management trained					
Yes	56 (14)	6 (2)		0	96.9
No	248 (62)	21 (7)		1 (1)	97.8

### Testing of socio demographic characteristics and the nurses’ responses to the action: “I check and assess the patient’s condition every time the alarm beeps”

The respondents scored highly on this question where the age group between 36 and 44 scored 93.2%, females scored 90.7%, KRN’s scored 100%, nurses who had worked for 10 years scored 91.3%, nurses who had worked specifically in the CCU for more than 10 years scored 94.2%, nurses that were CCN trained scored 90.6% and those that had not been trained in alarm management scored 91.1%.

### Testing of socio demographic characteristics and the nurses’ responses to the action: “I reset alarm settings of the machines each time I admit a patient”

The age group between 25 and 35 years scored 93.5%, both the males and the females scored 89%, the KRCHN’s scored 89.4%, the nurses who had worked for less than 2 years scored 100%, the nurses who had worked in CCU for more than 10 years scored 91.3%, the ones that were CCN trained scored 90.3% and the ones that had been trained in alarm management scored 89%. These were the highest scores.

## Discussion

The results from this study show that the respondents in this study usually respond to alarms of all durations, that is whether of short duration, frequently occurring or rare alarms. This therefore shows that the respondents in this study value the importance of alarms. In contrast to other studies where the findings indicate that nurses respond to alarms for different reasons, not just the fact that the alarm sounds. In a study conducted in the US nurses were found to adjust the order of their activities by evaluating alarm urgency in relation to the patients’ condition and had a greater tendency to react to alarms that beep for long and alarms that occur rarely as opposed to all the time. As workload complexity increases, alarm response and task performance deteriorates. Thus signal duration is an important influence to the nurses’ response but workload, patient condition and task complexity may lead to other reaction strategies [1].

Most of the respondents in this study reported that they do not fill alarm checklists and no alarm checklists are available in the unit and yet reported that documenting alarm parameters in the medical record was found to be an effective intervention for improving alarm adjustment compliance. This therefore shows that no alarm protocols are available in the institution as echoed by some of the respondents. The fact that no research has been undertaken on alarm management in the country also plays a role as it shows how little attention has been paid to alarm management.

On the various actions or nursing interventions that were posed to the respondents, the respondents reported that they carry out most of the interventions/actions. The respondents however scored 60% and above in some of the questions on alarm actions when the questions were tested against the social demographic factors. Since alarm management is very critical in the management of critically ill patients, it was expected that the scores would all be from around 90% to 100%. These scores therefore show that the staffs in the CCU need to be sensitized on the importance of alarm management. From the findings it was also noted that there was a statistically significant relationship between the nurses’ action of assessing the cause of the alarm beep and age. The nurses aged 36–44 years scored the highest as compared to those aged between 25 and 35 years and 45–55 years who more or else scored the same. This should probably be looked into in future studies. The younger nurses could be ignoring to assess the cause of the alarm beep because of the assumption that it is a false alarm and the older ones from experience could ignore because they can use the patient’s physiological status to detect whether they need to respond or not.

Health care personnel have been shown in previous studies to use alarms in a variety of ways depending on the particular process on which they are working. Sometimes when they appear not to have noticed the alarms, they may have in fact made use of the information and respond much later, perhaps after a minute or so [9].

## Conclusion

One of the conclusions drawn from this study is that the nurses do not fill alarm checklists and majority of them still manage to respond to alarms appropriately. Due to the scores the respondents received in this study, the researcher concludes that the respondents need training on alarm management and sensitization on the importance of alarm management.

The results of this study cannot be compared to any other studies nor be used to make concrete conclusions. In the future, observational comparison studies geared towards these particular variables should be undertaken to support whether any associations do exist.

Alarm protocols should also be established in the unit and the element of alarm checklists should be introduced. Finally more research should be undertaken on alarm management where all the nurses, doctors and biomedical personnel should be included.

## Additional file

Additional file 1: Data set for the Assessment of Nurses Interventions in the Management of Clinical Alarms in the Critical Care Unit. This data set contains all the data collected from a larger study conducted to assess the management of clinical alarms in the nursing care of critically ill patients at the critical care unit, KNH. Some of the variables captured were used to assess the other specific objectives in the larger study. For this particular manuscript the variables that are relevant are; the social demographic variables such as, age, gender, professional qualification, years that the participant has worked as a nurse, years that the participant has worked in CCU, whether the participant is trained in Critical Care Nursing, if the participant has been trained on alarm management, the number of hours of training and the year he or she was trained. Another variable captured in the dataset that is applicable in this manuscript is alarms that the nurses are more likely to respond to, number of nurses that fill alarm checklists and reasons as to why the nurses do not fill alarm checklists. Nursing interventions or actions in the management of clinical alarms are also variables that give insight into the findings in this manuscript. The interventions include; ensuring proper skin preparation before placement of electrodes, daily change of electrodes, assessing the cause of the alarm beep, disabling alarms every time they beep, pausing alarms every time they beep, re-setting alarm limits every time they beep, ignoring alarms every time they beep, checking and assessing the patient’s condition every time the alarm beeps and re-setting of alarms each time a patient is admitted. (XLSX 29 kb)

## Abbreviations

AACN: American Association of Critical Care Nurses
AARC: American Association for Respiratory Care
BScN: Bachelor of Science in Nursing
CCN: Critical Care Nursing
CCU: Critical Care Unit
CPD: Continuous Professional Development
ECG: Electrocardiogram
ERC: Ethics and Research Committee
ERC: Ethics and research Committee
KNH: Kenyatta National Hospital
KRCHN: Kenya Registered Community Health Nurse
KRN: Kenya Registered Nurse
MScN: Masters of Science in Nursing
UoN: University of Nairobi
